# Functional connectivity in dementia with Lewy bodies: A within‐ and between‐network analysis

**DOI:** 10.1002/hbm.23901

**Published:** 2017-11-29

**Authors:** Julia Schumacher, Luis R. Peraza, Michael Firbank, Alan J. Thomas, Marcus Kaiser, Peter Gallagher, John T. O'Brien, Andrew M. Blamire, John‐Paul Taylor

**Affiliations:** ^1^ Institute of Neuroscience, Newcastle University, Campus for Ageing and Vitality Newcastle upon Tyne NE4 5PL United Kingdom; ^2^ Interdisciplinary Computing and Complex BioSystems (ICOS) research group, School of Computing, Newcastle University Newcastle upon Tyne NE4 5TG United Kingdom; ^3^ Institute of Neuroscience, Newcastle University, The Henry Wellcome Building Newcastle upon Tyne NE2 4HH United Kingdom; ^4^ Department of Psychiatry University of Cambridge School of Medicine Cambridge CB2 0SP United Kingdom; ^5^ Institute of Cellular Medicine & Newcastle Magnetic Resonance Centre, Campus for Ageing and Vitality Newcastle upon Tyne NE4 5PL United Kingdom

**Keywords:** Alzheimer's disease, basal ganglia, dual regression, FSLNets, neurodegeneration, Parkinsonism, resting‐state networks

## Abstract

Dementia with Lewy bodies (DLB) is a common form of dementia and is characterized by cognitive fluctuations, visual hallucinations, and Parkinsonism. The phenotypic expression of the disease may, in part, relate to alterations in functional connectivity within and between brain networks. This resting‐state study sought to clarify this in DLB, how networks differed from Alzheimer's disease (AD), and whether they were related to clinical symptoms in DLB. Resting‐state networks were estimated using independent component analysis. We investigated functional connectivity changes in 31 DLB patients compared to 31 healthy controls and a disease comparator group of 29 AD patients using dual regression and FSLNets. Within‐network connectivity was generally decreased in DLB compared to controls, mainly in motor, temporal, and frontal networks. Between‐network connectivity was mainly intact; only the connection between a frontal and a temporal network showed increased connectivity in DLB. Differences between AD and DLB were subtle and we did not find any significant correlations with the severity of clinical symptoms in DLB. This study emphasizes the importance of reduced connectivity within motor, frontal, and temporal networks in DLB with relative sparing of the default mode network. The lack of significant correlations between connectivity measures and clinical scores indicates that the observed reduced connectivity within these networks might be related to the presence, but not to the severity of motor and cognitive impairment in DLB patients. Furthermore, our results suggest that AD and DLB may show more similarities than differences in patients with mild disease.

## 1 INTRODUCTION

Dementia with Lewy bodies (DLB) is a common form of degenerative dementia in older age and accounts for 4%–8% of all dementia cases clinically (Vann Jones and O'Brien, 2014). It is characterized by core symptoms of cognitive fluctuations, complex visual hallucinations, and Parkinsonism (McKeith et al., 2005) in contrast to Alzheimer's disease (AD) which is mainly characterized by memory loss, particularly in the early stages (Calderon, 2001). Neuroimaging methods such as resting‐state functional magnetic resonance imaging (fMRI) can aid in better understanding the underlying brain changes associated with DLB and how these differ from other dementia subtypes. Resting‐state fMRI can be used to study brain functional connectivity and enables characterization of resting‐state networks (RSNs) which are sets of brain regions that are spatially distinct, but show coordinated activity in the absence of a specific task (Biswal, Yetkin, Haughton, & Hyde, 1995; Lowe, Mock, & Sorenson, 1998). Several RSNs have been consistently found in healthy participant studies and involve brain regions that are related to different functions such as visual, motor and sensory processing, attention, salience, and memory (Damoiseaux et al., 2006). One resting‐state network that has been of particular interest is the default mode network (DMN) which is typically active during rest and deactivated upon the execution of a task (Raichle et al., 2001) and whose connectivity has been consistently found to be affected by AD (Binnewijzend et al., 2012; Greicius, Srivastava, Reiss, & Menon, 2004).

Most studies investigating functional connectivity in DLB have used seed‐based approaches (Galvin, Price, Yan, Morris, & Sheline, 2011; Kenny, Blamire, Firbank, & O'Brien, 2012; Kenny, O'Brien, Firbank, & Blamire, 2013) or only considered a small set of RSNs based on *a priori* hypotheses (Franciotti et al., 2013; Lowther, O'Brien, Firbank, & Blamire, 2014; Peraza et al., 2014); overall findings are somewhat inconsistent. While some studies have found that connectivity was generally decreased in DLB compared to age‐matched healthy controls (Lowther et al., 2014; Peraza et al., 2014), other studies only report increased connectivity in DLB compared to controls (Kenny et al., 2012; Kenny et al., 2013). Furthermore, the networks that have been found to be altered in DLB differ between studies. Decreased connectivity in DLB was reported for salience, executive (Lowther et al., 2014), frontoparietal, sensorimotor, and temporal networks (Peraza et al., 2014) whereas increased connectivity has been shown for basal ganglia (Kenny et al., 2013; Lowther et al., 2014) and thalamus (Kenny et al., 2013). In particular, the role of the DMN in DLB has been debated with different studies showing increased (Galvin et al., 2011; Kenny et al., 2012), decreased (Lowther et al., 2014) or unchanged connectivity within this network compared to controls (Franciotti et al., 2013; Peraza et al., 2014). In addition to reporting inconsistent findings, previous analyses have been limited to studying within‐network connectivity without considering connectivity changes between different RSNs. Therefore, the aim of this study was to investigate functional connectivity changes in DLB patients compared to healthy controls within and between a wide range of RSNs without *a priori* selection. We also included a disease comparator group of AD patients to investigate which changes in functional connectivity are specific to DLB (rather than dementia per se) and might help to differentiate it from other forms of dementia. We hypothesized to find changes in functional connectivity in DLB in the following networks: motor and basal ganglia networks because of previous evidence for their implication in Parkinsonism (Szewczyk‐Krolikowski et al., 2014), attentional networks based on previous results in DLB (Peraza et al., 2014) and the presence of a wide range of attentional deficits in DLB (Ballard et al., 2001), and possibly visual networks given DLB‐related impairments in visual processing (Mosimann et al., 2004). The second aim was to investigate whether the observed connectivity changes in DLB were related to the core clinical symptoms of visual hallucinations, cognitive fluctuations, and Parkinsonism to test if the present analysis could help in furthering our understanding of the etiological mechanisms underlying these symptoms in DLB.

## 2 METHODS

### 2.1 Participants

The study involved 102 participants who were over 60 years of age: 33 were diagnosed with probable DLB, 36 with probable AD, and 33 were age‐matched healthy controls (HC) with no history of psychiatric or neurological illness.

Participants from two contemporary independent studies conducted at one research center were combined for this analysis. Both studies recruited patients from the local community‐dwelling population who had been referred to old age psychiatry and neurology services, and were approved by the local ethics committee. DLB and AD diagnoses were performed independently by two experienced old‐age psychiatrists using consensus criteria for probable DLB (McKeith et al., 2005) and probable AD (McKhann, Drachman, Folstein, & Katzman, 1984; McKhann et al., 2011).

### 2.2 Data acquisition

MR imaging for both studies was performed on the same 3T Philips Intera Achieva scanner. The imaging protocol was the same in both studies except for a different resolution of the structural scans. To account for this, in the group analysis a dichotomous covariate of no interest for study membership was included. Structural images were acquired with a magnetization prepared rapid gradient echo (MPRAGE) sequence, sagittal acquisition, echo time 4.6 ms, repetition time 8.3 ms, inversion time 1250 ms, flip angle = 8°, SENSE factor = 2, and in‐plane field of view 256 × 256 mm^2^ with slice thickness 1.2 mm, yielding a voxel size of 0.93 × 0.93 × 1.2 mm^3^ (study 1) and in‐plane field of view 240 × 240 mm^2^ with slice thickness 1.0 mm, yielding a voxel size of 1.0 × 1.0 × 1.0 mm^3^ (study 2). Resting‐state scans for both studies were obtained with a gradient echo echo‐planar imaging sequence with 25 contiguous axial slices, 128 volumes, anterior–posterior acquisition, in‐plane resolution = 2.0 × 2.0 mm, slice thickness = 6 mm, repetition time = 3000 ms, echo time = 40 ms, and field of view = 260 × 260 mm^2^. DLB patients who were taking dopaminergic medication were scanned in the motor ON state.

### 2.3 Preprocessing

A first preprocessing step was carried out using FEAT (FMRI Expert Analysis Tool) Version 6.0 which is part of the FMRIB's software library (FSL, http://www.fmrib.ox.ac.uk/fsl) including motion correction using FMRIB's Linear Image Registration Tool (MCFLIRT), slice‐timing correction, and spatial smoothing with a 6.0mm full width at half maximum Gaussian kernel. Participants were excluded if the MCFLIRT‐estimated motion parameters exceeded 2 mm translation and/or 2° rotation. To assess differences in movement between the three groups due to patients with Parkinsonian symptoms the following formula was used (Liao et al., 2010):

$$\text{head}\;\text{motion}/\text{rotation}={\left(M-1\right)}^{-1}\sum_{i=2}^{M}\sqrt{{\left|x_{i}-x_{i-1}\right|}^{2}-{\left|y_{i}-y_{i-1}\right|}^{2}-{\left|z_{i}-z_{i-1}\right|}^{2}},$$

where *M* is the total number of volumes (*M* = 128) and *x_i_*, *y_i_*, and *z_i_* are the translations/rotations at the *i*th time point in *x*, *y*, and *z* direction.

Denoising was performed with ICA‐AROMA in FSL which performs single‐subject independent component analysis (ICA) to remove motion components from each participant's functional data (Pruim, Mennes, Buitelaar, & Beckmann, 2015a; Pruim et al., 2015b). Additionally, eroded CSF and white matter masks were estimated using FAST in FSL and the mean signal inside the mask was regressed out of each participant's cleaned functional data. Functional and structural images were then co‐registered using boundary based registration in FSL, and normalized to the standard MNI template using Advanced Normalization Tools (Avants et al., 2011; Klein et al., 2009). Finally, functional data were temporally high‐pass filtered with a cutoff of 150 s and resampled to a resolution of 4 × 4 × 4 mm^3^. Grey matter probability maps were obtained from the FAST‐segmented T1 images and included as voxel‐wise spatial covariates in the group comparison analyses.

### 2.4 Analysis of resting‐state data

**Figure 1:**
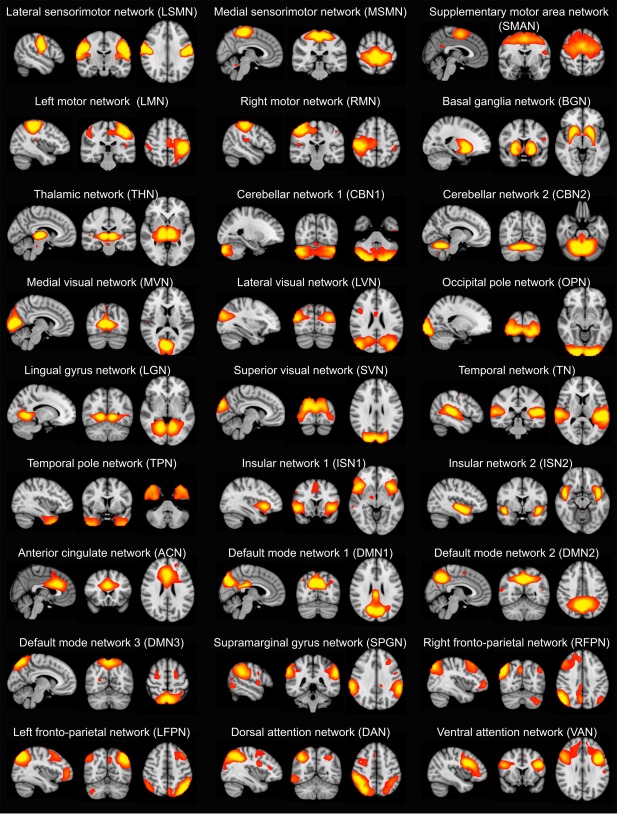
Spatial maps of the 27 resting‐state networks (RSNs) obtained from the independent healthy control group. RSN maps are thresholded at 3 < *z* < 12. Images are shown in radiological convention, that is, the left side of the image corresponds to the right hemisphere [Color figure can be viewed at http://wileyonlinelibrary.com]

Resting‐state networks were estimated using an independent set of 42 HC participants from two previous studies that were conducted on the same MR scanner with similar imaging protocols (see Section 1 of the Supporting Information for more information). The temporally concatenated data from all additional control participants were subjected to a group‐ICA using FSL's MELODIC (Multivariate Exploratory Linear Optimized Decomposition into Independent Components). To obtain more reliable components, a meta ICA approach was adopted as in (Biswal et al., 2010; Poppe et al., 2013). Briefly, MELODIC was repeated 25 times on randomized subsets of 30 out of the 42 HC participants. Subsequently, a meta ICA run was performed on the concatenated components from all individual ICA runs. A model order of 70 independent components was chosen for the individual as well as the meta ICA as this has been shown to be optimal for assessing disease‐related group differences (Abou Elseoud et al., 2011; Dipasquale et al., 2015). To identify reliable components, the spatial correlation of each meta component across the individual ICA runs was calculated and components with a correlation <0.6 across runs were excluded (Cerliani et al., 2015). Furthermore, the meta ICA procedure was repeated using all HC participants from the main analysis and compared to the components from the independent group to ensure that the selected RSNs were present in both cohorts. All meta ICA components from the independent cohort that survived these reliability checks were visually inspected with respect to their spatial maps (Kelly et al., 2010) and 27 were identified as being of biological interest according to the previous literature (Agosta et al., 2012; Beckmann, DeLuca, Devlin, & Smith, 2005; Damoiseaux et al., 2008) (Figure 1 and Supporting Information, Table S2).

Subsequently, FSL‐dual regression was run with all 27 identified RSNs concatenated in a single 4D image, to obtain subject‐specific representations of the RSN spatial maps and associated subject‐specific time courses. Group differences between DLB and HC and between DLB and AD were assessed using FSL's randomize function with 10,000 permutations and family‐wise error correction for multiple comparisons using threshold‐free cluster enhancement (TFCE). Covariates of no interest were included to control for age, gender, and study membership. Additionally, in order to reduce the impact of cortical atrophy differences between our participant groups, we also included grey matter probability maps as voxel‐wise regressors in the linear model (Damoiseaux, Prater, Miller, & Greicius, 2012).

To investigate between‐network connectivity, the FSLNets package was applied to the subject‐specific time series from dual regression (http://fsl.fmrib.ox.ac.uk/fsl/fslwiki/FSLNets). Full and partial correlations were calculated between all pairs of RSNs and the resulting correlation coefficients were converted to *z* scores for further analysis. Partial correlations are computed as correlations between two RSNs while controlling for the effect of all other RSNs and are thought to reflect more direct connections (Smith et al., 2011). FSL‐randomize with 10,000 permutations was then applied to assess group differences in between‐network connectivity including covariates for age, gender, and study membership. Results were FWE corrected for multiple comparisons.

### 2.5 Statistical analyses

Statistical analyses were carried out in IBM SPSS version 23. Table 1 shows which statistical tests were applied to assess between‐group differences for the different clinical variables. Spearman's rank correlation was used to assess relations between functional connectivity and clinical scores in the DLB patients, including the three scores related to the core DLB symptoms (CAF total score for cognitive fluctuations, UPDRS III for Parkinsonism, and NPI hallucination subscale which was specifically focused on visual hallucination occurrence) and a measure of global cognition (MMSE). Correlations were computed for the mean connectivity within clusters with significant differences between DLB and controls (from dual regression) and for between‐network connectivity scores for connections with significant between‐group differences (from FSLNets). All correlations were computed in the DLB group separately.

**Table 1:** Demographic and clinical variables, mean (standard deviation)

	HC (*N* = 31)	AD (*N* = 29)	DLB (*N* = 31)	Between‐group differences
Male:Female	22:9	20:9	19:12	χ^2^ = 0.73, *p* = 0.70^a^
Study 1:Study 2	15:16	13:16	12:19	χ^2^ = 0.60, *p* = 0.74^a^
Age	76.4 (7.2)	75.2 (8.6)	78.13 (6.7)	F_2,88_ = 1.16, *p* = 0.32^b^
AChEI	‐	26	28	χ^2^ = 0.007, *p* = 0.93^c^
PD meds	‐	1	18	χ^2^ = 20.66, *p* < 0.001^c^
Duration	‐	3.7 (1.7)^f^	3.4 (2.3)	*U* = 339, *p* = 0.14^d^
MMSE	28.9 (1.1)	21.8 (3.8)	22.03 (4.3)	t_58_ = 0.20, *p* = 0.85^e^
CAMCOG	96.7 (3.2)	70.3 (13.5)	73.29 (13.6)	t_58_ = 0.86, *p* = 0.39^e^
UPDRS III	1.94 (2.8)	3.5 (4.0)	18.1 (10.2)	t_58_ = 7.32, *p* < 0.001^e^
CAF total	‐	1.00 (2.51)^f^	4.8 (4.9)^g^	t_56_ = 3.66, *p* = 0.001^e^
NPI total	‐	5.9 (5.5)^h^	14.55 (11.03)^i^	t_54_ = 3.68, *p* = 0.001^e^
NPI hall	‐	0^j^	1.6 (1.8)^i^	t_53_ = 4.53, *p* < 0.001^e^

*Note*. AChEI, number of patients taking acetylcholinesterase inhibitors; AD, Alzheimer's disease; CAF total, Clinical Assessment of Fluctuations total score; CAMCOG, Cambridge Cognitive Examination; DLB, dementia with Lewy bodies; Duration, duration of cognitive symptoms in years; HC, healthy controls; Mayo total, Mayo Fluctuations Scale; Mayo cognitive, Mayo Fluctuation cognitive subscale; Mayo arousal, Mayo Fluctuations arousal subscale; MMSE, Mini Mental State Examination; PD meds, number of patients taking dopaminergic medication for the management of Parkinson's disease symptoms; UPDRS III, Unified Parkinson's Disease Rating Scale III (motor subsection); NPI, Neuropsychiatric Inventory; NPI hall, NPI hallucination subscore.

a Chi‐square test HC, AD, DLB.

b One‐way ANOVA HC, AD, DLB.

c Chi‐square test AD, DLB.

d Mann–Whitney *U* test AD, DLB.

e Student's *t*‐ test AD, DLB.

f *N* = 28.

g *N* = 30.

h *N* = 27.

i *N* = 29.

j *N* = 26.

## 3 RESULTS

One AD patient had to be excluded due to coregistration errors. Additionally, two HC, six AD, and two DLB participants were excluded because of excessive motion. This resulted in 31 DLB patients, 29 AD patients, and 31 healthy controls for further analysis. The overall motion for all included participants was not significantly different between the three groups (Kruskal–Wallis test; rotation, H_2_ = 1.93, *p* = .38; translation, H_2_ = 1.13, *p* = .57).

### 3.1 Demographics

All three groups were matched for age and gender and the two dementia groups were matched in terms of overall cognition (MMSE and CAMCOG) and duration of dementia (Table 1). As expected, the number of patients taking dopaminergic medication was significantly higher in the DLB group. The number of patients taking cholinesterase inhibitors was not significantly different between the dementia groups. DLB patients were significantly more impaired in terms of Parkinsonism, visual hallucinations, and cognitive fluctuations than the AD patients.

### 3.2 Within‐network connectivity

Between‐group comparisons of the dual regression results were performed across the whole brain space, that is, they were not spatially bounded by the thresholded RSN spatial maps shown in Figure 1.

**Figure 2:**
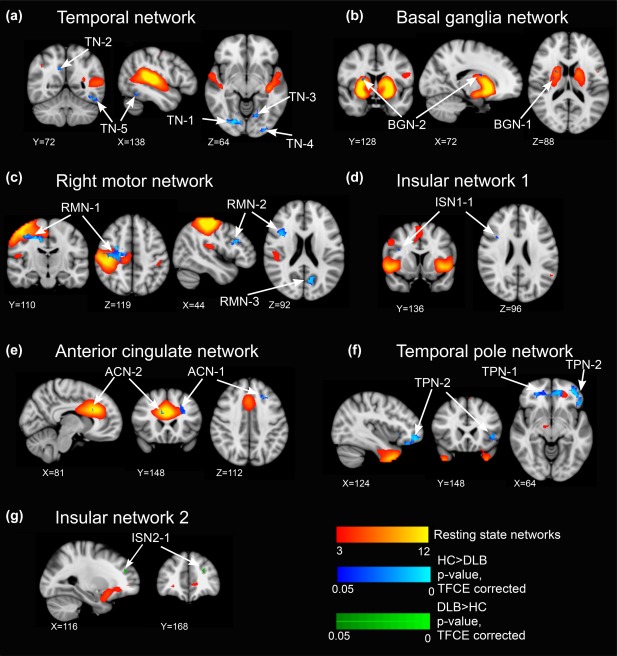
Dual regression results for comparison between DLB and HC. RSN maps are shown in red‐yellow. (a–f) Clusters with decreased connectivity in DLB; HC > DLB, *p* < .05, threshold free cluster enhancement (TFCE) corrected, shown in blue. (g) Clusters with increased connectivity in DLB; DLB > HC, *p* < .05, TFCE corrected, shown in green. See Table 2 for more information on cluster locations and sizes. All images are shown in radiological convention [Color figure can be viewed at http://wileyonlinelibrary.com]

Decreased connectivity in DLB compared to controls was observed for nine RSNs including the lateral sensorimotor network, the medial sensorimotor network, the temporal network, the basal ganglia network, the right motor network, the thalamic network, the insular network 1, the anterior cingulate network, and the temporal pole network. Increased connectivity in DLB compared to controls was found in very small clusters for the left motor network, the ventral attention network, and the insular network 2 (Figure 2, Table 2, and Supporting Information, Figure S2).

**Table 2:** Dual regression results

	*N* voxels	*p* value	MNI (*X*, *Y*, *Z*)	Location
*HC > DLB*				
Lateral sensorimotor network				
LSMN‐1	1	0.046	24, 28, 30	L supplementary motor cortex
Medial sensorimotor network				
MSMN‐1	1	0.048	26, 21, 19	L hippocampus, white matter
Temporal network				
TN‐1	34	0.002	17, 12, 16	R lingual gyrus, R occipital fusiform gyrus
TN‐2	20	0.014	21, 21, 26	R posterior cingulate gyrus, R precuneus
TN‐3	10	0.02	26, 15, 15	L lingual gyrus
TN‐4	9	0.017	30, 8, 16	L inferior lateral occipital cortex
TN‐5	6	0.007	34, 18, 14	L inferior temporal gyrus
TN‐6	5	0.033	33, 11, 13	L inferior lateral occipital cortex
TN‐7	2	0.043	34, 14, 23	L superior lateral occipital cortex
TN‐8	1	0.040	37, 17, 13	L inferior temporal gyrus
Basal ganglia network				
BGN‐1	5	0.039	15, 29, 21	R putamen
BGN‐2	2	0.035	17, 32, 22	R caudate
Right motor network				
RMN‐1	142	0.001	15, 26, 30	R precentral gyrus
RMN‐2	54	0.003	14, 34, 24	R middle frontal gyrus, R inferior frontal gyrus
RMN‐3	22	0.007	25, 15, 23	L precuneus
Thalamic network				
THN‐1	5	0.039	30, 9, 24	L superior lateral occipital cortex
Insular network 1				
ISN1‐1	1	0.032	13, 34, 24	R inferior frontal gyrus
Anterior cingulate network				
ACN‐1	11	0.028	29, 37, 24	L superior frontal gyrus, L middle frontal gyrus
ACN‐2	4	0.044	20, 37, 25	R anterior cingulate cortex
ACN‐3	1	0.027	34, 18, 15	L inferior temporal gyrus
Temporal pole network				
TPN‐1	190	0.005	24, 40, 19	R anterior cingulate cortex, L anterior cingulate cortex, R paracingulate, L paracingulate
TPN‐2	100	0.003	31, 44, 16	L frontal pole, L inferior frontal gyrus, L frontal orbital cortex
TPN‐3	3	0.041	21, 22, 30	R precuneus, R precentral gyrus
*DLB > HC*				
Left motor network				
LMN‐1	4	0.012	16, 26, 31	R precentral gyrus, white matter
Ventral attention network				
VAN‐1	1	0.036	27, 16, 22	L precuneus
Insular network 2				
ISN2‐1	6	0.021	29, 42, 24	L frontal pole
*AD > DLB*				
No significant clusters				
*DLB > AD*				
Default mode network 1				
DMN1‐1	1	0.044	13, 12, 24	R superior lateral occipital cortex
DMN1–2	1	0.025	13, 12, 27	R superior lateral occipital cortex

All clusters are reported with *p* < .05, threshold free cluster enhancement (TFCE) corrected. The table shows the number of significant voxels per cluster, the minimal *p* value inside the cluster, the MNI coordinates of the voxel with minimal *p* value, and the location of the cluster (estimated from the Harvard–Oxford Cortical and Subcortical Structural Atlases and the Cerebellar Atlas in FSL).

Very small clusters of increased connectivity in DLB compared to AD were found for the default mode network 1 (Table 2 and Supporting Information, Figure S2). There were no clusters of decreased connectivity in DLB compared to AD.

There were no significant differences in connectivity between DLB patients who were taking dopaminergic medication (*N* = 18) compared to those who were not (*N* = 13) except for two very small clusters of increased connectivity in the medicated patients comprising one voxel for the supplementary motor area network in left frontal orbital cortex and right superior frontal gyrus. A comparison between patients on and off cholinesterase inhibitors was not possible due to small numbers in the latter group.

### 3.3 Between‐network connectivity

**Figure 3:**
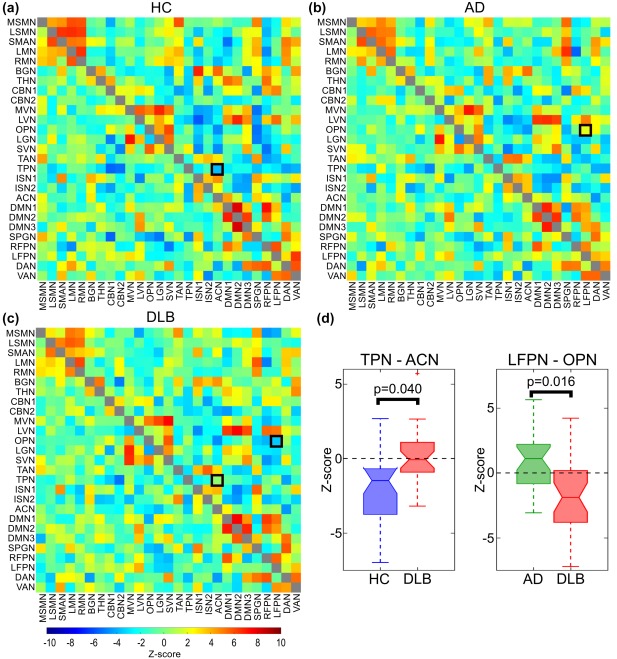
Correlation matrices from FSLNets analysis for (a) HC, (b) AD, and (c) DLB. Upper triangular matrices show full correlations while partial correlations are plotted in the lower triangular matrices. (d) Boxplots show *z* scores for edges with significant group differences for full correlations (black squares in panel a–c, *p* < .05, FWE corrected for multiple comparisons). OPN, occipital pole network; LFPN, left fronto‐parietal network; TPN, temporal pole network; ACN, anterior cingulate network [Color figure can be viewed at http://wileyonlinelibrary.com]

When considering full correlations, there was a change in connectivity between the temporal pole and the anterior cingulate networks in DLB compared to HC (Figure 3). While this connection showed a negative correlation in controls, the mean correlation was around zero in the DLB group. There were no connections with decreased connectivity in DLB compared to controls.

When comparing AD and DLB a significant difference was found for the connection between the left fronto‐parietal and the occipital pole networks which were positively correlated in the AD group, but showed a negative correlation in DLB (Figure 3). There were no significant differences for either contrast when using partial correlations.

### 3.4 Exploratory correlations with clinical scores in dementia with Lewy bodies

After applying FDR correction for multiple comparisons we did not find any significant correlations between clinical scores and mean within‐network connectivity in the DLB group for the clusters that showed significant group differences. Uncorrected results are shown in Supporting Information, Table S4. As an additional exploratory analysis, we also investigated voxelwise correlations between clinical scores and connectivity within the clusters resulting from the group comparison (see Section 5 of the Supporting Information). However, even with this more granular analysis we did not find any significant correlations after applying FDR correction for multiple comparisons.

## 4 DISCUSSION

We investigated within‐ and between‐network connectivity in a wide range of RSNs in DLB compared to healthy controls as well as AD patients. With respect to within‐network connectivity more decreases than increases in connectivity were identified in the DLB group compared to controls, mainly in motor, temporal, and frontal networks. This is the first study to investigate how connectivity between different RSNs is affected by DLB. However, the results from this analysis suggest that long‐range functional connections are largely intact in DLB as there was only one connection between a frontal and a temporal network that showed altered between‐network connectivity compared to controls. When directly comparing both dementia groups we only found very small differences indicating that AD and DLB might not be that different with respect to their resting‐state functional connectivity. Furthermore, we did not find any consistent relation between altered connectivity in DLB and any clinical variables suggesting that this analysis method might not be the most suitable to identify neural correlates of clinical DLB symptoms.

### 4.1 Decreased connectivity in motor networks in dementia with Lewy bodies

Connectivity was decreased in DLB compared to controls in several motor networks, including both sensorimotor, the basal ganglia, and the right motor networks. Overall, the observed changes in these networks correspond well to the clinical manifestation of DLB which is—among other core symptoms—characterized by Parkinsonian motor features (McKeith et al., 2005). Moreover, the results show substantial overlap with previous findings in Parkinson's disease (PD) and emphasize the significance of alterations in motor networks in DLB even though primarily this condition is characterized by cognitive decline and, frequently, significant AD co‐pathology (Irwin et al., 2017).

Decreased connectivity in the basal ganglia network has been found in PD compared to controls and AD and has been suggested as a biomarker for early PD (Rolinski et al., 2015; Szewczyk‐Krolikowski et al., 2014). While we found similar results in our DLB group, the clusters of decreased connectivity were much smaller than in previous PD studies. This might be due to the use of dopaminergic medication in many of our DLB patients which has been shown to restore basal ganglia connectivity to near‐normal levels (Szewczyk‐Krolikowski et al., 2014). The present results stand in contrast to previous studies in DLB that found increased basal ganglia connectivity compared to controls (Kenny et al., 2013; Lowther et al., 2014). The discrepancy between previous results in DLB and the present results and more recent PD studies is likely to be due to the use of different preprocessing methods, especially with respect to the removal of motion artefacts. It has recently been argued that motion correction approaches such as those used in previous DLB studies might have led to spurious findings and that prior results might have to be re‐evaluated using more appropriate motion correction techniques such as those applied in this study (Ciric et al., 2017; Parkes, Fulcher, Yucel, & Fornito, 2017; Power, Schlaggar, & Petersen, 2015). This is especially crucial when studying elderly patients and comparing groups with different degrees of motor symptoms (van Dijk, Sabuncu, & Buckner, 2012).

In addition to decreased basal ganglia connectivity we found reduced connectivity within cortical motor networks. The right motor network showed large clusters of decreased connectivity in DLB within primary motor areas. Sensorimotor networks have been commonly shown to be altered in Lewy body diseases (Tessitore, Giordano, de Micco, Russo, & Tedeschi, 2014; Wu et al., 2011; Yu, Liu, Wang, Chen, & Liu, 2013) and lower connectivity within the motor cortex has been reported previously in DLB (Peraza et al., 2016, 2014; Taylor, Colloby, McKeith, & O'Brien, 2013). In addition to reduced connectivity within the motor network itself we found that cognitive control areas, such as frontal and default mode areas, were less strongly involved in this network in DLB, which might be related to impairments of voluntary movement control in this disease group. However, we did not find any correlations between the reduction in motor network connectivity and the severity of Parkinsonism. It might be that motor connectivity changes are related to the presence of Parkinsonian symptoms, but not their severity.

### 4.2 DLB‐related changes in nonmotor networks

With respect to nonmotor networks, we found decreased connectivity in DLB compared to controls mainly in temporal and frontal networks. The temporal network showed a general disconnection from different occipital regions which agrees with previous findings in DLB (Peraza et al., 2014; Taylor et al., 2012). The connections between occipital and temporal cortices represent the ventral visual stream which is involved in object recognition (Ungerleider and Haxby, 1994). A breakdown of this important visual pathway might thus be related to visuo‐perceptual difficulties in DLB (Mosimann et al., 2004). However, similarly to previous studies we did not find any significant correlations with frequency or severity of visual hallucinations (Peraza et al., 2014). As was previously posited, it may be that the observed connectivity changes foster a cortical state that is permissive for the occurrence of visual hallucinations, but that is not directly related to their severity of frequency of occurrence.

The temporal pole network demonstrated lower synchronizations in DLB compared to controls, mainly in frontal areas such as anterior cingulate cortex (ACC) and frontal pole. Similarly, the frontal anterior cingulate network showed a disconnection from inferior temporal regions. The observed reduced involvement of the ACC within the temporal pole network in DLB seemed to be compensated by an increase in between‐network connectivity between the temporal pole and the anterior cingulate networks. The ACC is an important region involved in cognitive control and emotional processing (Bush, Luu, & Posner, 2000) and abnormalities in this region have been associated with different aspects of Lewy body diseases. While reduced metabolism in the ACC has been found in both DLB and PD with dementia (Yong, Yoon, An, & Lee, 2007), synaptic and pathological changes in this region have been implicated in visual hallucinations in DLB (Teaktong et al., 2005) and cognitive deficits in PD (Kövari et al., 2003). The present results provide further evidence for the importance of ACC abnormalities in Lewy body diseases and suggest that the previously described changes at the synaptic level might lead to more wide‐range disruptions of the functional connectivity profile of this region.

However, whilst we replicated the common finding of decreased DMN connectivity in the posterior cingulate cortex in AD (Supporting Information, Figure S1 and Table S3; Binnewijzend et al., 2012; Greicius et al., 2004), we did not find any changes in DMN connectivity in DLB compared to controls. Additionally, DMN connectivity was increased in DLB compared to AD albeit only in very small clusters. These results indicate that the finding of DMN hypoactivity is rather specific to AD and might not be present in DLB patients (Franciotti et al., 2013; Peraza et al., 2014).

The results of this study suggest that long‐range connections are largely intact in DLB which is somewhat contradictory to results from a previous graph‐based analysis that found a relative loss of medium and long range connections in DLB (Peraza, Taylor, & Kaiser, 2015). However, while this study focuses on spatially distinct networks, the previous graph‐theoretic approach is a more global analysis. It might thus be that connections between independent resting‐state networks are rather intact while this might not be true for long distance connections in general.

### 4.3 Comparison of the dementia groups

In contrast to previous studies we did not find large differences between the two dementia groups with respect to their within‐network functional connectivity (Galvin et al., 2011; Lowther et al., 2014). An important difference to previous studies was the use of a more stringent motion correction technique and the inclusion of a covariate to control for voxel‐wise grey matter differences. Previous studies on AD‐DLB differences did not include a grey matter covariate even though grey matter loss is generally more severe in AD than in DLB (Watson, O'Brien, Barber, & Blamire, 2012) and might thus lead to spurious results in a group comparison (Damoiseaux et al., 2012). Furthermore, it has been shown that subtle differences in motion between groups can be mistaken for neuronal effects (van Dijk et al., 2012).

In our investigation, however, we found a between‐network connectivity difference between AD and DLB for the left frontoparietal and occipital pole networks, which showed opposed synchronizations; positive in AD and negative in DLB. In the HC group, the correlation between these two networks is on average negative, which suggests that the positive correlation seen in the AD group is likely to represent an abnormal shift of connectivity from negative to positive correlation. Functional alterations in occipital and attentional systems have been previously reported in AD (Li et al., 2012; Sorg et al., 2007) although not between these two systems. Further research will be needed to corroborate their altered functional inter‐relations.

### 4.4 Limitations

One limitation of this study is that some of the DLB patients were on dopaminergic medication and scanned in the ON state which might have influenced their functional connectivity measures. However, it has been shown that dopaminergic medication tends to normalize connectivity towards healthy levels (Szewczyk‐Krolikowski et al., 2014; Tahmasian et al., 2015), which implies that the group differences that we found were not due to medication. Another possible limitation is the fact that all diagnoses were based on clinical assessment rather than pathological confirmation. However, it has been shown that the standardized clinical criteria used in this study show high specificity when validated against autopsy findings (McKeith et al., 2000).

### 4.5 Conclusion

Functional differences between AD and DLB were subtle and suggest that these two dementias may have more similarities than differences in patients with mild disease. Additionally, our study revealed a general decrease in functional connectivity in DLB compared to healthy aging in motor, frontal, and temporal networks with a relative sparing of the DMN. The observed functional connectivity alterations might be related to the presence of motor and cognitive impairment in DLB as networks commonly associated with these functions showed lower connectivity. However, we were not able to find significant correlations between decreased functional connectivity in these RSNs and clinical scores associated with motor and cognitive function in DLB. Further research will be needed to infer the neural mechanisms associated with the symptomatic complexity of DLB and its differences with AD.

## Supporting information

Additional Supporting Information may be found online in the supporting information tab for this article.

Supporting Information
