# Supplementary material for: Functional connectivity in dementia with Lewy bodies: A within‐ and between‐network analysis
Source: Hum Brain Mapp. 2017 Nov 29;39(3):1118–29. doi: 10.1002/hbm.23901 (PMC5900719; doi:10.1002/hbm.23901)
Supplement: Supplementary file 1 — Supporting Information [file HBM-39-1118-s001.pdf]

Supplementary Material: Functional connectivity in dementia with Lewy bodies: A within- and between-network analysis

Julia Schumacher, Luis R. Peraza, Michael Firbank, Alan J. Thomas, Marcus Kaiser, Peter Gallagher, John T. O'Brien, Andrew M. Blamire, John-Paul Taylor

Contents

1. Independent healthy control group
2. Locations of RSN spatial maps
3. Comparison of functional connectivity between HC and AD
4. Positive and negative correlations
5. Clinical correlations in the DLB group
6. Voxel-based morphometry analysis
7. Effect of dichotomous study covariate
8. Supplementary references

1. Independent healthy control group

Table S1: Demographics of independent healthy control group, compared to control group from main analysis

	HC main analysis (N=31)	HC for RSN template estimation (N=42)	Between-group comparison
Male: female	22:9	25:17	χ^2=1.02, p=0.31
Age	76.4 (7.2)	69.0 (8.7)	t_{70}=3.85, p<0.001
MMSE	28.9 (1.1)	29.2 (1.4)	t_{70}=1.08, p=0.29

HC, healthy controls; MMSE, Mini Mental State Examination

To estimate independent healthy resting state networks (RSNs), 44 healthy older adult controls (HC) from two previous studies were selected. They were significantly younger than the HCs from the main analysis, but matched in terms of overall cognition (Supplementary Table S1).

All participants were scanned on the same scanner as the participants from the main analysis. Eighteen of the additional HC participants were scanned with a slightly different scanner protocol with a change in the TR to 2072 ms and a change in the voxel size of the resting state scans to 3 x 3 x 4 mm^3.

The resting state data were preprocessed in the same way as described in the main manuscript. Two subjects were excluded because they exceeded the motion exclusion criteria resulting in 42 independent HC participants that were included in the generation of the RSN templates.

2. Locations of RSN spatial maps

Table S2: List of all resting state networks (RSNs) included in the analysis. Anatomical labels refer to bilateral areas if not stated otherwise. Locations of RSNs are estimated from the Harvard-Oxford Cortical and Subcortical Structural Atlases and the Cerebellar Atlas in FSL.

RSN name		Brain regions
Lateral sensorimotor network	LSMN	Pre- and postcentral gyrus
Medial sensorimotor network	MSMN	Pre- and postcentral gyrus, supplementary motor area
Supplementary motor area network	SMAN	Supplementary motor area, precentral gyrus
Left motor network	LMN	Left post- and precentral gyrus
Right motor network	RMN	Right post- and precentral gyrus
Basal ganglia network	BGN	Putamen, caudate
Thalamic network	THN	Thalamus
Cerebellar network 1	CBN1	Cerebellum crus I, crus II
Cerebellar network 2	CBN2	Cerebellum V, VI
Medial visual network	MVN	Intracalcarine cortex, supracalcarine cortex, lingual gyrus
Lateral visual network	LVN	Superior lateral occipital cortex, precuneus
Occipital pole network	OPN	Occipital pole
Lingual gyrus network	LGN	Lingual gyrus, intracalcarine cortex
Superior visual network	SVN	Superior lateral occipital cortex, occipital pole
Temporal network	TN	Planum temporale, Heschl’s gyrus
Temporal pole network	TPN	Temporal pole
Insular network 1	ISN1	Insular cortex, frontal operculum cortex
Insular network 2	ISN2	Insular cortex, planum polare
Anterior cingulate network	ACN	Anterior cingulate cortex
Default mode network 1	DMN1	Precuneus, posterior cingulate cortex
Default mode network 2	DMN2	Precuneus
Default mode network 3	DMN3	Precuneus, superior lateral occipital cortex
Supramarginal gyrus network	SPGN	Supramarginal gyrus
Right fronto-parietal network	RFPN	Right superior lateral occipital cortex, right angular gyrus, right middle frontal gyrus, left superior lateral occipital cortex
Left fronto-parietal network	LFPN	Left superior lateral occipital cortex, right angular gyrus, left middle frontal gyrus, right superior lateral occipital cortex
Dorsal attention network	DAN	Superior parietal lobule, supramarginal gyrus, superior lateral occipital cortex
Ventral attention network	VAN	Middle frontal gyrus, inferior frontal gyrus

3. Comparison of functional connectivity between HC and AD

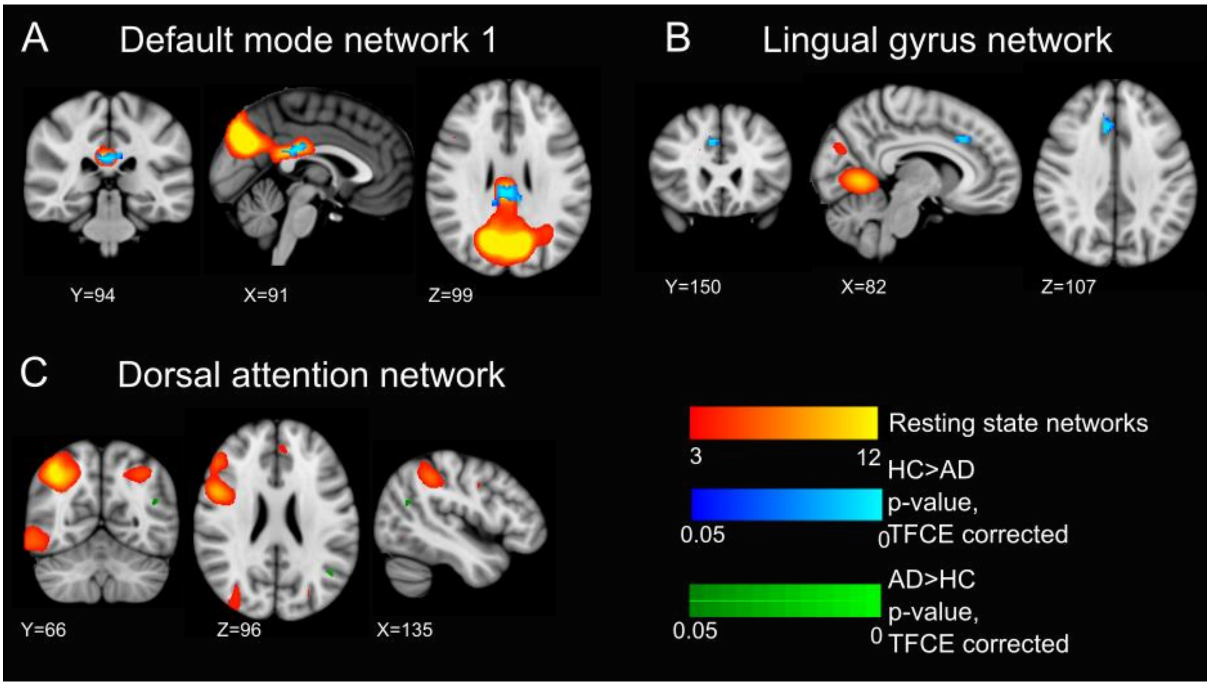

Figure S1: Dual regression results for comparison between AD and HC. RSN maps are shown in red-yellow**.** A,B) Clusters with decreased connectivity in AD; HC>AD, $p<0.05$, threshold free cluster enhancement (TFCE) corrected, shown in blue. C) Cluster with increased connectivity in AD; AD>HC, $p<0.05$, TFCE corrected, shown in green.

Table S3: Dual regression results for comparison between AD and HC. All clusters are reported with $p<0.05$, threshold free cluster enhancement (TFCE) corrected. The table shows the number of significant voxels per cluster, the minimal p-value inside the cluster, the MNI coordinates of the voxel with minimal p-value, and the location of the cluster (estimated from the Harvard-Oxford Cortical and Subcortical Structural Atlases and the Cerebellar Atlas in FSL).

	N voxels	p-value	MNI (X, Y, Z)	Location
HC > AD				
Default mode network 1				
DMN1-1	63	<0.001	20, 22, 24	L posterior cingulate, R posterior cingulate
Lingual gyrus network				
LGN-1	17	0.002	20, 37, 27	R paracingulate gyrus
Right motor network				
RMN-1	2	0.028	12, 33, 24	R precentral gyrus, R inferior frontal gyrus
AD > HC				
Dorsal attention network				
DAN-1	6	0.03	34, 17, 24	L angular gyrus

4. Positive and negative correlations

Although decreased connectivity in the DLB group is reported for all clusters in panels A-F of Figure 2, it was evident that some of these results were due to correlations shifting from positive in the control group to negative in the DLB group (e.g. TN-1). Similarly, increased connectivity in the DLB group could also be due to correlations being negative in HC, and shifting to positive correlations in DLB (e.g. ISN2-1), see Supplementary Figure S2.

Anticorrelations are not easy to interpret and shifts from positive to negative or from negative to positive correlations in patient groups are even harder to understand. However, while it has previously been believed that anticorrelations might be a mere result of certain preprocessing steps [Murphy et al., 2009], it has recently been argued that they have an actual biological origin [Chai et al., 2012; Keller et al., 2013; Liang et al., 2012]. Furthermore, negative synchronizations have been observed in Parkinson’s disease where it has been hypothesized that they might represent a compensatory mechanism [Peraza et al., 2017].

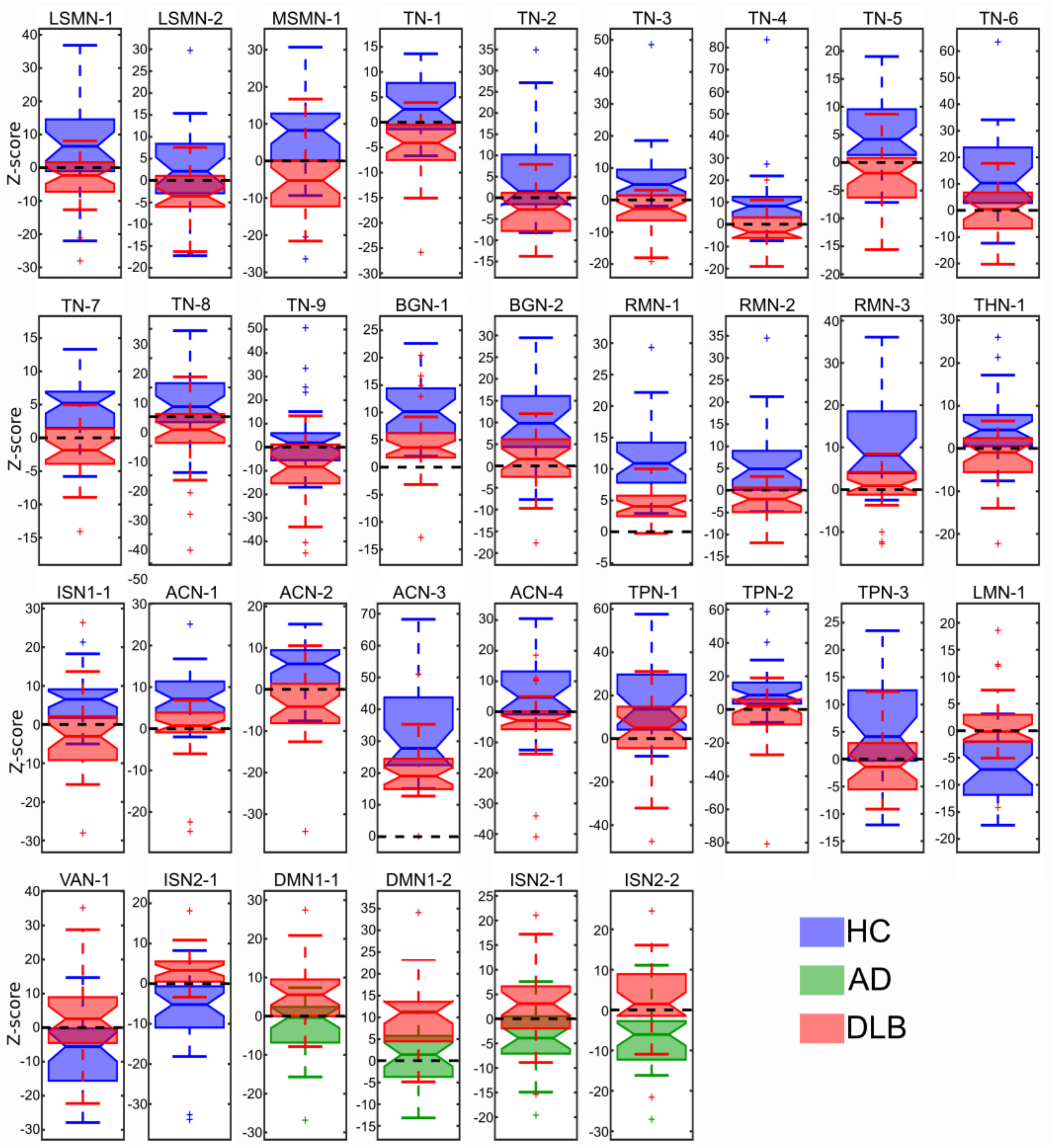

Figure S2: Mean z scores for all clusters showing a significant difference between DLB and HC and DLB and AD (see Figure 2 and Table II). In each boxplot the central line corresponds to the sample median, the upper and lower border of the box represent the 25th and 75th percentile, respectively, and the length of the whiskers is 1.5 times the interquartile range, outliers are shown by +.

5. Clinical correlations in the DLB group

Table S4: Spearman's rank correlation between mean functional connectivity within significant clusters from dual regression and clinical scores in the DLB group. All clusters are shown that have an uncorrected p-value<0.05.

	CAF total[a]		
		p, uncorrected	p, FDR-corrected
ACN-4	ρ = -0.38	0.04	0.931
DMN1-2	ρ = -0.37	0.047	0.931
	CAF duration[a]		
ACN-4	ρ = -0.40	0.03	0.931
DMN1-2	ρ = -0.39	0.03	0.931
ISN2-1	ρ = 0.38	0.04	0.931
	CAF frequency[a]		
TN-6	ρ = -0.37	0.04	0.931
	NPI hallucinations[b]		
LSMN-2	ρ = -0.44	0.02	0.931

[a] N = 30, [b] N=29

In addition to investigating correlations with mean connectivity within a cluster, we also tested voxelwise correlations with clinical scores. To this end, the dual regression z-scores for all DLB participants were concatenated in one 4D image and correlations with clinical scores were tested using a GLM in FSL with the respective clinical score as covariate in the design matrix. Statistical significance was assessed using randomize with 5000 permutations including a mask for the significant clusters from the HC-DLB and AD-DLB group comparisons. There was one cluster of 4 voxels in the right occipital fusiform gyrus belonging to the temporal network (TN-1) that showed negative correlation with the CAF total score. Additionally, there was a very small cluster of one voxel in the right precentral gyrus belonging to the right motor network (RMN-1) where connectivity was positively correlated with the CAF total score. However, none of these clusters survived FDR-correction for multiple comparisons. We did not find any significant clusters for any of the other clinical scores.

6. Voxel-based morphometry analysis

To study changes in grey matter between the three groups a voxel-based morphometry analysis was conducted using DARTEL in SPM12 using age, gender, and total intracranial volume as covariates. The AD group showed clusters of reduced grey matter compared to controls, mainly in right and left hippocampus (Supplementary Figure S3). No regions showed increased grey matter in AD compared to controls.

The DLB group had reduced grey matter in a small cluster in right cingulate (Supplementary Figure S4). Again, there were no areas of increased grey matter in DLB compared to controls. There was also no difference in grey matter between the two dementia groups.

HC>AD

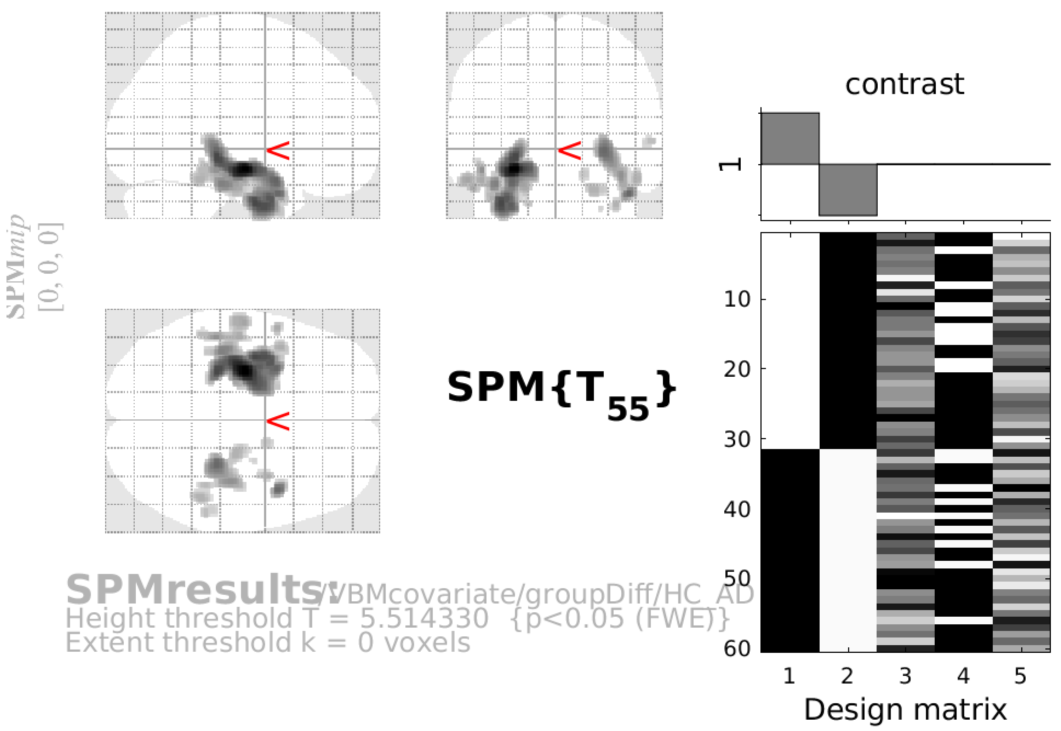

Statistics: ***p-values adjusted for search volume***

set-level		cluster-level				peak-level					mm mm mm
p	c	$p_{FWE-corr}$	$q_{FDR-corr}$	k_E	p_{uncorr}	$p_{FWE-corr}$	$q_{FDR-corr}$	T	$(Z_{\equiv})$	p_{uncorr}	
0.000	9	0.000	0.000	4588	0.000	0.000	0.004	8.27	6.64	0.000	-27 -15 -14
						0.000	0.028	7.33	6.10	0.000	-27 -32 -8
						0.000	0.028	7.30	6.08	0.000	-36 -8 -34
		0.001	0.024	112	0.010	0.000	0.033	6.96	5.87	0.000	44 6 -28
		0.000	0.000	1127	0.000	0.001	0.034	6.92	5.85	0.000	28 -32 -3
						0.002	0.116	6.47	5.56	0.000	39 -24 -18
						0.008	0.253	6.09	5.30	0.000	48 -42 -20
		0.007	0.156	32	0.138	0.006	0.242	6.18	5.36	0.000	-54 6 -26
		0.000	0.012	149	0.004	0.008	0.253	6.09	5.30	0.000	22 -20 -27
		0.003	0.098	57	0.055	0.009	0.268	6.06	5.28	0.000	36 0 -36
		0.004	0.121	46	0.081	0.015	0.354	5.91	5.17	0.000	57 -39 3
		0.007	0.156	33	0.133	0.018	0.406	5.84	5.13	0.000	15 -2 -16
		0.025	0.488	7	0.488	0.032	0.641	5.66	5.00	0.000	26 -9 -38

table shows 3 local maxima more than 8.0mm apart

Height threshold: T = 5.51, p = 0.000 (0.050)
Extent threshold: k = 0 voxels
Expected voxels per cluster, <k> = 15.298
Expected number of clusters, <c> = 0.05
FWEp: 5.514, FDRp: 6.923, FWEc: 7, FDRc: 112

Degrees of freedom = [1.0, 55.0]
FWHM = 13.3 13.7 13.7 mm mm mm; 8.9 9.1 9.1 {vox
Volume: 5241928 = 1553164 voxels = 2044.0 resels
Voxel size: 1.5 1.5 1.5 mm mm mm; (resel = 738.30 v

Figure S3: Results from VBM analysis comparing AD and controls.

HC > DLB

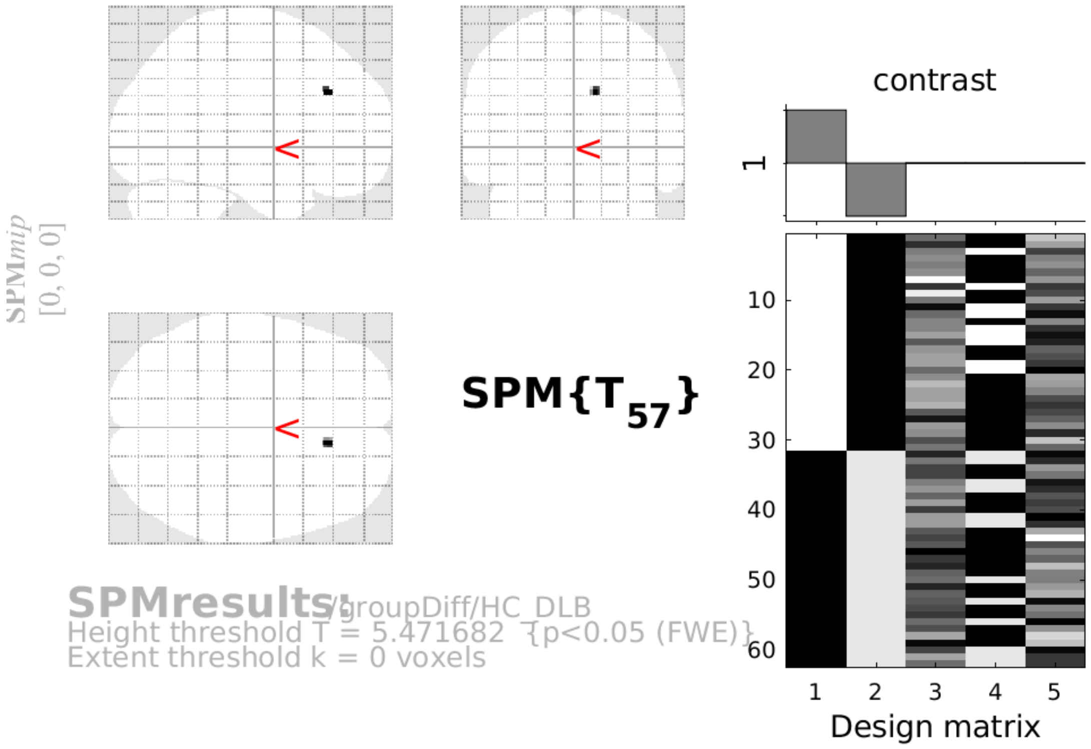

Statistics: ***p-values adjusted for search volume***

	cluster-level				peak-level					mm mm mm
	$p_{FWE\text{-}corr}$	$q_{FDR\text{-}corr}$	k_E	p_{uncorr}	$p_{FWE\text{-}corr}$	$q_{FDR\text{-}corr}$	T	$(Z_{\equiv})$	p_{uncorr}	
	0.020	0.397	11	0.397	0.023	0.461	5.71	5.06	0.000	10 30 30

table shows 3 local maxima more than 8.0mm apart

Height threshold: T = 5.47, p = 0.000 (0.050)
Extent threshold: k = 0 voxels
Expected voxels per cluster, <k> = 16.465
Expected number of clusters, <c> = 0.05
FWEp: 5.472, FDRp: Inf, FWEc: 11, FDRc: Inf

Degrees of freedom = [1.0, 57.0]
FWHM = 13.5 14.0 13.9 mm mm mm; 9.0 9.3 9.2 {vox
Volume: 5252043 = 1556161 voxels = 1946.8 resels
Voxel size: 1.5 1.5 1.5 mm mm mm; (resel = 776.67 v

Figure S4: Results from VBM analysis comparing DLB and controls.

7. Effect of dichotomous study covariate

To study the effect of the inclusion of the dichotomous study covariate, we repeated the dual regression analysis without the covariate and compared the results. Group differences were found in the same networks (see Supplementary Figure S5), the only difference being that some clusters became a bit larger (e.g. basal ganglia network) or smaller (e.g. insular networks) when including the covariate. Nevertheless, we decided to include the covariate in our main analysis to account for small differences between the two studies in terms of recruitment and imaging protocol.

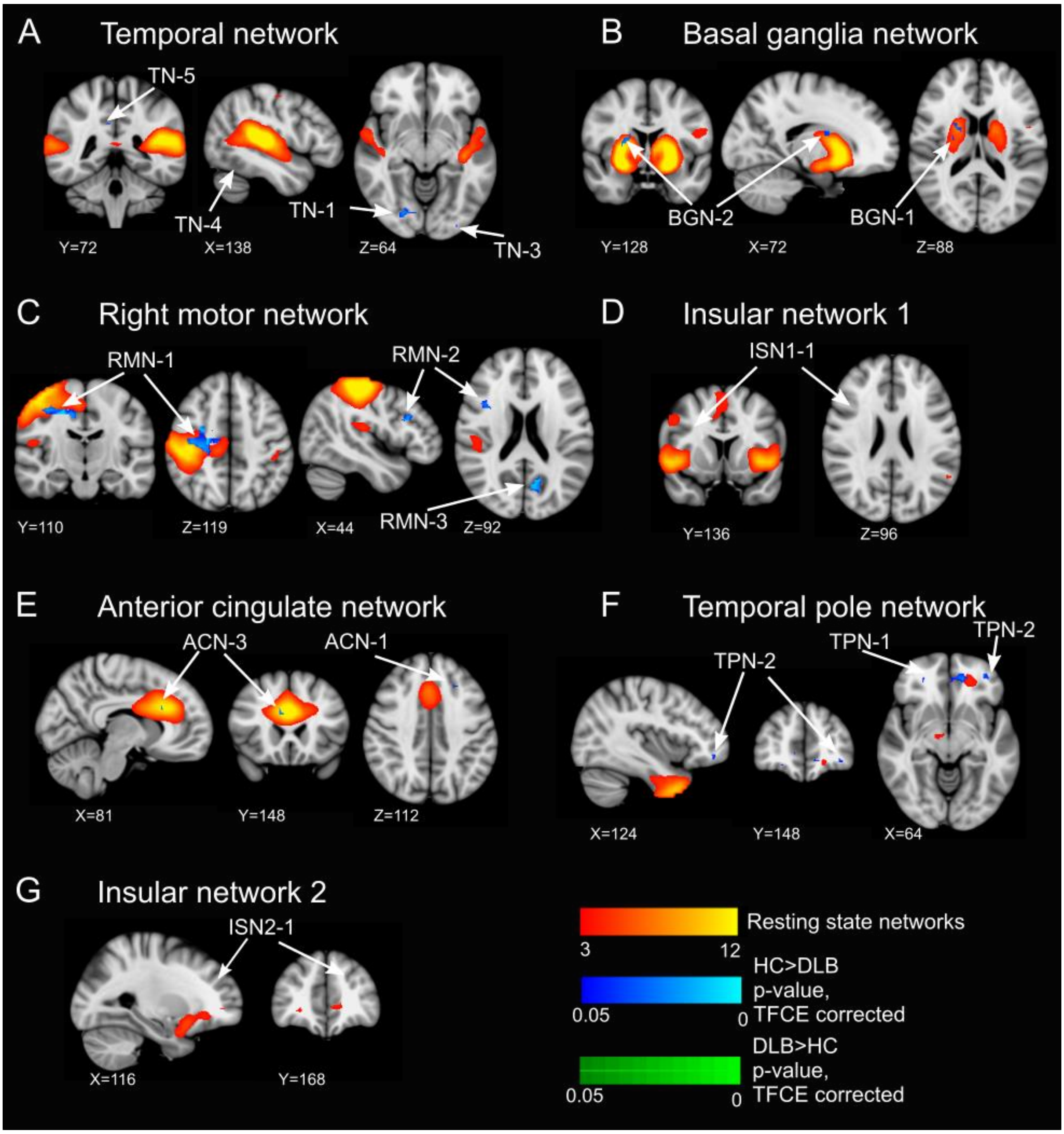

Figure S5: Dual regression results for comparison between DLB and HC without the addition of a dichotomous covariate for study membership. RSN maps are shown in red-yellow and significant group differences are shown in blue/green. All images shown in radiological convention.

8. Supplementary references

Chai XJ, Castañán AN, Öngür D, Whitfield-Gabrieli S (2012): Anticorrelations in resting state networks without global signal regression. Neuroimage 59:1420–1428.

Keller CJ, Bickel S, Honey CJ, Groppe DM, Entz L, Craddock RC, Lado FA, Kelly C, Milham M, Mehta AD (2013): Neurophysiological Investigation of Spontaneous Correlated and Anticorrelated Fluctuations of the BOLD Signal. J Neurosci 33:6333–6342.

Liang Z, King J, Zhang N (2012): Anticorrelated resting-state functional connectivity in awake rat brain. Neuroimage 59:1190–1199.

Murphy K, Birn RM, Handwerker DA, Jones TB, Bandettini PA (2009): The impact of global signal regression on resting state correlations: Are anti-correlated networks introduced? Neuroimage 44:893–905.

Peraza LR, Nesbitt D, Lawson RA, Duncan GW, Yarnall AJ, Khoo TK, Kaiser M, Firbank MJ, O'Brien JT, Barker RA, Brooks DJ, Burn DJ, Taylor J-P (2017): Intra- and inter-network functional alterations in Parkinson's disease with mild cognitive impairment. Hum Brain Mapp 38:1702–1715.
